# ^1^H NMR-Based Metabolomics and Machine Learning Reveal Candidate Metabolic Markers Associated with *Coffea arabica* Genealogical Groups

**DOI:** 10.3390/foods15142498

**Published:** 2026-07-15

**Authors:** Marcelo R. Malta, Gladyston R. Carvalho, Denis H. S. Nadaleti

**Affiliations:** 1 Experimental Field of Lavras, Minas Gerais Agricultural Research Agency (EPAMIG), Lavras 37200-900, MG, Brazil; carvalho@epamig.br; 2 Department of Agriculture, Federal University of Lavras (UFLA), Lavras 37200-900, MG, Brazil; denisnadaleti@ufla.br

**Keywords:** ^1^H NMR metabolomics, chemometrics, genealogical discrimination, chemical fingerprinting, machine learning

## Abstract

The genetic diversity of *Coffea arabica* L. plays a strategic role in the development of cultivars with differentiated agronomic and sensory attributes. However, the chemical discrimination of genealogical groups still represents a challenge due to the strong environmental influence on bean metabolism. In this study, ^1^H NMR-based metabolomics associated with supervised chemometric models was applied to discriminate three genealogical groups of *C. arabica* (Bourbon, Mundo Novo, and Timor Hybrid) using 38 green coffee samples harvested during the 2020 and 2021 crop seasons. Spectra were processed by bucketing and analyzed using PCA, PLS-DA, Elastic Net, Random Forest, and SVM-RBF. PCA explained 52.91% of the total variability in the first two components but did not promote clear separation among groups. Among the supervised classifiers, PLS-DA showed the best balance between predictive performance, statistical stability, and chemical interpretability, achieving a balanced accuracy of 89.4% under repeated cross-validation. The five-component model presented an overall error rate of 12.1% after repeated 5-fold cross-validation with 100 simulations. VIP scores and loadings identified discriminant metabolites for each group: lipids, amino acids, and organic acids for Bourbon; amino acids and organic acids for Mundo Novo; and chlorogenic acids for Timor Hybrid. These results highlight the potential of ^1^H NMR metabolomics as a complementary tool for varietal discrimination and support for breeding programs.

## 1. Introduction

Coffee is one of the most consumed beverages worldwide and represents one of the major agricultural commodities, with Brazil being the largest global producer and exporter. The species *Coffea arabica* L. accounts for approximately 60% of global coffee production and exhibits high genetic diversity, expressed in traditional cultivars such as Bourbon, Typica, and Mundo Novo, as well as interspecific hybrids such as Timor Hybrid (HdT) [1,2]. This diversity represents a strategic resource for breeding programs, particularly in response to the increasing demand for cultivars with higher productivity, resistance to biotic and abiotic stresses, and differentiated beverage quality attributes [3,4].

Conventional methods for varietal characterization, including physicochemical analyses and sensory evaluations, present limitations due to overlapping characteristics among cultivars [2,5]. In this context, metabolomics has emerged as a promising tool for identifying chemical signatures associated with genotype, environment, and post-harvest processing. Different analytical approaches, such as mass spectrometry and nuclear magnetic resonance (NMR), have been applied to coffee to discriminate geographical origins, cultivation conditions, and sensory attributes [6,7].

^1^H NMR stands out because of its rapidity, non-destructive nature, and ability to generate comprehensive metabolic profiles in a single analysis [8]. When combined with chemometric techniques, it enables the identification of discriminant metabolites and the development of predictive classification models. Nevertheless, studies applying NMR associated with machine learning algorithms to discriminate genealogical groups of *C. arabica* are still scarce. We hypothesized that ^1^H NMR metabolomic fingerprints contain sufficiently stable metabolic information to discriminate *Coffea arabica* genealogical groups despite environmental variability.

Chemometric analysis of spectroscopic data generally relies on two complementary families of pattern-recognition methods. Unsupervised methods, such as Principal Component Analysis (PCA) and Hierarchical Cluster Analysis (HCA), do not use class information and are mainly exploratory, being employed to reduce dimensionality and to detect natural groupings, trends, and outliers. Their main advantage is the absence of modeling bias, whereas their limitation is that the captured variance does not necessarily correspond to the information of interest, since it may be dominated by environmental or processing factors rather than by genotype. Supervised methods, such as Partial Least Squares Discriminant Analysis (PLS-DA), Elastic Net, Random Forest, and Support Vector Machines (SVM), use class labels to build predictive models and typically provide greater discriminatory power; however, they require rigorous validation to avoid overfitting, particularly when the number of spectral variables greatly exceeds the number of samples [8,9]. The combination of these algorithms with ^1^H NMR fingerprints has been increasingly applied to coffee authentication, species and varietal discrimination, and geographical origin assessment, providing both classification models and chemically interpretable markers [6,7,10,11,12]. It must be emphasized, however, that the metabolome represents a phenotype resulting from genotype × environment (G × E) interactions rather than a direct readout of the genetic background [3,13]; consequently, the metabolic patterns associated here with genealogical groups should be interpreted as candidate chemical markers modulated by both genetic and environmental factors, and not as pure genotypic signatures.

The accurate classification of coffee genealogical groups is crucial to ensure quality in the specialty coffee market, guarantee authenticity, and support breeding programs [4]. The Bourbon cultivar originated from introductions of *C. arabica* from Yemen to Reunion Island (formerly Bourbon) during the eighteenth century and served as the genetic basis for several subsequent cultivars. Mundo Novo originated from a natural cross between Bourbon and Typica identified in 1943 in São Paulo State, Brazil, and was later selected by the Agronomic Institute of Campinas (IAC). In Brazil, the Arabica coffee cultivar ‘Mundo Novo’ is one of the most widely planted cultivars due to its high yield, vigor, and stability [14]. Timor Hybrid resulted from a natural interspecific cross between *C. arabica* and *C. canephora*, first identified on Timor Island. From this lineage, derivatives such as Catimor (HdT × Caturra) and Sarchimor (HdT × Villa Sarchí) were developed and are widely used in breeding programs due to the incorporation of disease resistance genes [15].

However, environmental factors, including altitude, climatic conditions, agronomic management, and post-harvest processing, introduce metabolic variations and covariances that may mask signals associated with the genetic background in the coffee metabolome, making varietal discrimination based exclusively on chemical profiles difficult [10,11,13,16]. Integrative strategies combining genomic, metabolomic, and chemometric data have been proposed to increase the robustness of varietal discrimination and specialty coffee traceability, allowing the identification of chemical signatures associated with genetic background and beverage quality [10,12]. Despite recent advances in the application of metabolomics to coffee, few studies have investigated whether metabolomic signatures obtained by NMR remain sufficiently stable to discriminate genealogical groups of *Coffea arabica* under the strong environmental covariance associated with crop season, climate, altitude, and agronomic management. This limitation still represents one of the major challenges for the application of metabolomics to varietal discrimination and genetic traceability in coffee.

In this context, the present study aimed to apply ^1^H NMR-based metabolomics associated with exploration, multivariate analyses and supervised classifiers to evaluate the separation of three genealogical groups of *C. arabica* and identify potential chemical markers with applications in breeding programs and specialty coffee certification.

## 2. Materials and Methods

### 2.1. Study Site

The Active Germplasm Bank of Minas Gerais (BAG) is located at the Patrocínio Experimental Field of the Minas Gerais Agricultural Research Agency (EPAMIG), at 18°59′26″ S latitude, 48°58′95″ W longitude, and 975 m altitude, in the Alto Paranaíba region, Minas Gerais, Brazil. The soil is classified as a dystrophic Red-Yellow Latosol in an area with predominantly flat topography and slight slope. The regional climate is classified as subtropical mesothermal with rainy summers, dry winters, and hot summers (Cwa), according to the Köppen classification.

### 2.2. Harvesting, Processing, Drying, and Storage

Coffee harvesting was performed manually by strip picking on cloth, selecting predominantly ripe fruits (cherry stage). The fruits were subjected to dry processing to obtain natural coffee. Drying was carried out on suspended mesh trays with periodic turning until the beans reached a moisture content between 11 and 12% (wet basis). Subsequently, the dried coffee cherries were stored in a cold chamber at 18 °C for 30 days to homogenize moisture content. After this period, the samples were hulled and prepared for analysis.

### 2.3. Sample Identification

The experimental set consisted of 38 coffee samples (Table 1) belonging to three genealogical groups: Bourbon, Mundo Novo, and Timor Hybrid (HdT). Samples were collected during two crop seasons (2020 and 2021). The genealogical authenticity of the samples was ensured by their controlled provenance: all accessions originate from the Active Germplasm Bank (BAG) of Minas Gerais, maintained by EPAMIG at the Patrocínio Experimental Field, where each accession has a documented pedigree and genealogical record under the technical curation of the coffee breeding program. The genealogical classification was therefore based on institutional registration and pedigree documentation, and was not inferred from the chemical data themselves. All accessions were grown under the same edaphoclimatic conditions at a single site, and harvesting (selective picking at the cherry stage), processing, and drying were standardized and supervised by the institution’s technical staff.

### 2.4. Sample Preparation for NMR

Sample preparation followed a microextraction protocol. One hundred milligrams of ground green coffee were weighed using an analytical balance (AR2140, OHAUS, Parsippany, NJ, USA) and extracted with 650 μL of CD_3_OD containing 0.01% TMS (trimethylsilane) as an internal reference. Deuterated methanol (CD_3_OD) was selected as the extraction solvent because it provides broad metabolite coverage of green coffee extracts in a single acquisition, efficiently solubilizing polar and medium-polarity metabolites (sugars, amino acids, organic acids, chlorogenic acids, trigonelline, and caffeine) while still extracting the more apolar lipid fraction (free fatty acids and the methyl/methylene resonances of triacylglycerols observable in the δ 0.6–2.5 ppm region). This solvent is widely adopted in ^1^H NMR metabolomic protocols for coffee because it combines extraction efficiency with spectral reproducibility and a deuterium lock signal. The samples were homogenized using a vortex mixer for 1 min, subjected to sonication in an ultrasonic bath for 10 min at room temperature, and subsequently centrifuged (10.000 rpm for 5 min). An aliquot of 500 μL of the supernatant was transferred to 5 mm NMR tubes for spectroscopic analysis.

### 2.5. Acquisition and Processing of ^1^H NMR Spectra

The spectra were acquired using a Bruker Avance III spectrometer (600 MHz, 14.1 T; Bruker BioSpin, Rheinstetten, Germany) equipped with a 5 mm TCI cryoprobe. The zgpr pulse sequence was used for suppression of the residual water signal. Typical acquisition parameters included a relaxation delay (D1) of 20 s, acquisition time (AQ) of 2.72 s, 32 scans, TD of 64k points, and a spectral width of 12 kHz. Analyses were performed at 298 K in triplicate.

Spectral processing was carried out using TopSpin^®^ 3.1 software (Bruker BioSpin, Rheinstetten, Germany). Exponential multiplication (line broadening) of 0.3 Hz was applied, followed by manual phase correction and automatic baseline correction. Chemical shift referencing was performed at δ 0.00 ppm (TMS signal). Two-dimensional experiments (HSQC and HMBC) were performed on a representative sample for structural confirmation of metabolites.

### 2.6. Spectral Preprocessing

Multivariate preprocessing was performed using AMIX^®^ software (version 3.9.12). Regions corresponding to solvent signals (water, δH 5.00–4.60; methanol, δH 3.33–3.28) were excluded. The spectra were segmented (bucketing) into 0.02 ppm intervals, and the resulting matrix was exported for statistical analysis in R. Statistical analyses were conducted in the R environment (version 4.3.0) using customized routines specifically developed for this study. No proprietary chemometric software (e.g., MATLAB) was used; all classification algorithms (PLS-DA, Elastic Net, Random Forest, and SVM-RBF) and the resampling and validation procedures (nested cross-validation, repeated stratified cross-validation, GroupKFold, and the permutation test) were implemented in R (version 4.4.1; R Foundation for Statistical Computing, Vienna, Austria) using the caret (version 7.0.1), mixOmics (version 6.28.0), glmnet (version 4.1.10), randomForest (version 4.7.1.2), and e1071 (version 1.7.16) packages.

Before multivariate analyses, spectral data were standardized using Z-score scaling, meaning that each variable was centered by its meaning and scaled by its standard deviation (calculated from the training set within each cross-validation procedure). This scaling ensured comparability among variables in Principal Component Analysis and classification models. Each sample was represented by the mean of three replicates, and to avoid information leakage, grouped cross-validation was applied (GroupKFold with five folds). The spectral intensities (425 ^1^H NMR buckets) were centered and scaled before model fitting.

### 2.7. Statistical and Chemometric Analyses

Model performance was evaluated using Overall Accuracy (proportion of correctly classified samples) and Balanced Accuracy, defined as the average sensitivity (recall) across classes. The sensitivity of a class was calculated as the proportion of samples from that class correctly identified by the model. Balanced accuracy provided a performance measure adjusted for class imbalance (for example, Timor Hybrid contained more samples than Mundo Novo). Aggregated confusion matrices were generated by combining predictions from all cross-validation folds to inspect correct and incorrect classifications for each model.

#### 2.7.1. Exploratory Analysis

Initially, Principal Component Analysis (PCA) was performed as an unsupervised exploratory approach to evaluate the intrinsic variability of the samples and identify possible clustering patterns among genealogical groups. Before analysis, spectral data were autoscaled (mean-centered and divided by the standard deviation of each variable).

#### 2.7.2. Supervised Classification Models

Supervised classification models, including Partial Least Squares Discriminant Analysis (PLS-DA), Elastic Net, Random Forest (RF), and Support Vector Machine with radial basis function kernel (SVM-RBF), were evaluated for discrimination against *Coffea arabica* genealogical groups.

#### 2.7.3. Model Validation and Statistical Robustness

The statistical validation of the supervised models was structured into complementary stages, each with a specific purpose within the analytical workflow, avoiding overlap among the employed procedures. Initially, model hyperparameter optimization was performed using nested cross-validation. In this procedure, the inner loop was used exclusively for selecting the optimal hyperparameters of each algorithm, including the number of latent components in the PLS-DA model while the outer loop was used to estimate predictive performance on data not used during model training, thereby reducing optimistic bias and the risk of overfitting.

After defining the optimal hyperparameters, the final performance of the models was estimated using repeated stratified 5-fold cross-validation with 100 repetitions. In each repetition, approximately 80% of the samples were used for training and 20% for external validation, while maintaining the original proportion among genealogical groups. The metrics reported in Table 2 (overall accuracy, balanced accuracy, and Cohen’s Kappa coefficient) correspond to the mean values obtained from this repeated cross-validation procedure.

Additionally, the GroupKFold procedure was employed only as a complementary strategy to control information leakage associated with correlated samples originating from the same experimental crop season. In this case, samples belonging to the same experimental group were simultaneously kept either in the training or validation set, preventing indirect information sharing between folds. The results obtained using GroupKFold showed behavior consistent with those observed in repeated stratified cross-validation, reinforcing the robustness of the models.

Finally, the statistical significance of the PLS-DA model was independently evaluated using a permutation test (1000 permutations). In this procedure, genealogical class labels were randomly shuffled while maintaining the spectral structure of the dataset unchanged. The performance of the real model was then compared with the null distribution generated by the permuted models, allowing verification of whether the observed discrimination was significantly better than random classification. Thus, each validation procedure employed in this study had a specific and complementary role: nested cross-validation for model optimization, repeated stratified 5-fold cross-validation for predictive performance estimation, GroupKFold for controlling experimental dependence, and permutation testing for evaluating the statistical significance of the observed metabolomic discrimination.

## 3. Results

### 3.1. Exploratory Metabolomic Variability Among Coffea arabica Genealogical Groups

#### 3.1.1. Representative ^1^H NMR Spectra

**Figure 1:**
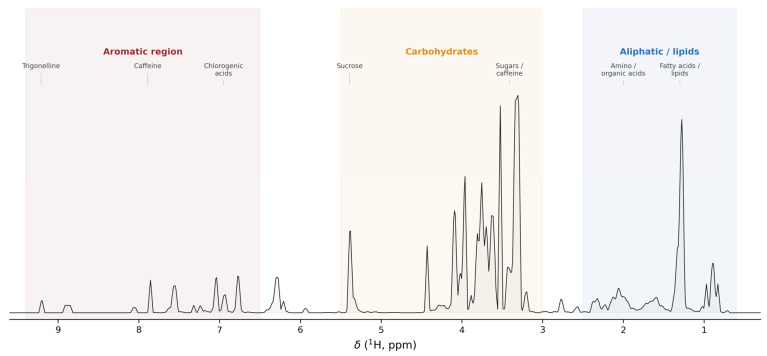
Representative ^1^H NMR spectrum (600 MHz, CD_3_OD) of a green coffee extract, highlighting the aliphatic/lipid (δ 0.6–2.5 ppm), carbohydrate (δ 3.0–5.5 ppm), and aromatic (δ 6.5–9.4 ppm) regions, with the main metabolite assignments (trigonelline, caffeine, chlorogenic acids, sucrose, sugars, amino/organic acids, and fatty acids/lipids). All samples showed a closely similar profile irrespective of genealogical group, which precluded discrimination by visual inspection alone (subsequent figures renumbered accordingly).

A representative ^1^H NMR spectrum of a green coffee extract is shown in Figure 1. All samples presented a very similar overall spectral profile regardless of genealogical group or crop season, reflecting the common metabolic background of *C. arabica*; consequently, the genealogical groups could not be distinguished by simple visual inspection of the spectra, which justified the use of multivariate analysis. Three main regions can be highlighted: (i) the aliphatic region (δ 0.6–2.5 ppm), dominated by the methyl and methylene resonances of fatty acids and lipids and by aliphatic amino acids and organic acids; (ii) the carbohydrate region (δ 3.0–5.5 ppm), associated with sucrose, other sugars, and caffeine N–CH_3_ signals; and (iii) the aromatic region (δ 6.5–9.4 ppm), corresponding to chlorogenic acids, trigonelline, and caffeine. The differences in the relative intensities of these regions among genealogical groups were subtle and are therefore better resolved by the supervised models described below, which provide a chemical rationale for the classification results, rather than by direct visual comparison of the spectra.

Although the overall spectral profiles were highly similar, subtle differences could be observed in specific spectral regions. Samples later classified as Bourbon tended to show relatively higher intensities in the carbohydrate region (δ 3.0–5.5 ppm), particularly in signals attributed to sucrose, as well as in parts of the aliphatic region associated with lipids. In contrast, Timor Hybrid samples exhibited relatively stronger resonances in the aromatic region (δ 6.5–9.4 ppm), especially those assigned to chlorogenic acids and caffeine-related compounds. Mundo Novo samples generally showed intermediate spectral characteristics, consistent with their intermediate position observed in the multivariate models. These differences were not sufficiently pronounced to allow visual discrimination of the genealogical groups but became evident after multivariate modeling using PCA and PLS-DA.

#### 3.1.2. Principal Component Analysis (PCA)

**Figure 2:**
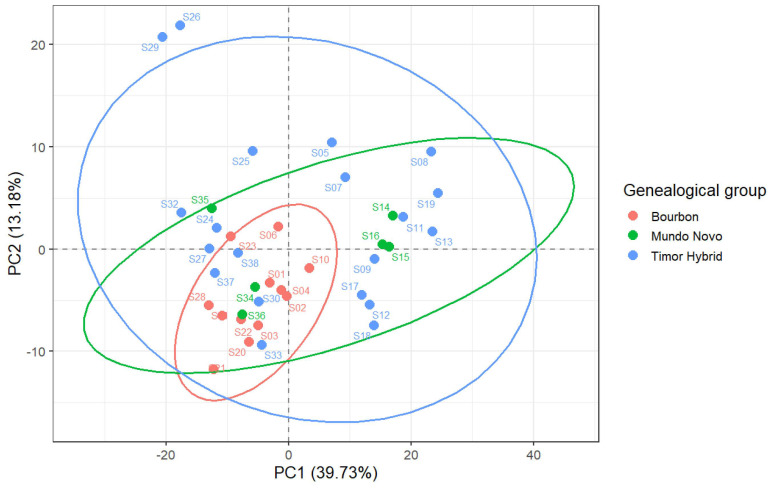
Dispersion of samples along the first two principal components (PC1 vs. PC2) after Z-score scaling. PC1 explained 39.73% of the variance and mainly separated Bourbon (low PC1 scores) from Timor Hybrid (high PC1 scores). PC2 explained an additional 13.18% of the variance. Bourbon and Timor Hybrid formed partially distinct groups, whereas Mundo Novo occupied an intermediate region, with some overlap still observed among the genealogical groups.

Principal Component Analysis (PCA) was performed to reduce data dimensionality and allow visualization of sample distribution according to the first two principal components (PC1 and PC2), which concentrated most of the variability. Exploratory PCA analysis of the ^1^H NMR dataset (Figure 2) revealed the intrinsic complexity of the classification problem. Although a tendency for grouping along PC1 and PC2 was observed, the genealogical groups showed substantial overlap in the PCA space. The cumulative variance explained by the first two components (PC1 ≈ 39.73%; PC2 ≈ 13.18%) totaled only 52.91% of the overall variance.

In the generated score plot, each point represents one sample, and colors indicate the different coffee genealogical groups. Part of the separation among groups could be visualized along PC1, indicating that this axis concentrated the greatest distinction among the analyzed chemical profiles. The absence of well-defined clusters in this low-dimensional space indicates that most spectral variance was not dominated by genealogical signals but rather by metabolic variations introduced by Genotype × Environment interaction (G × E). This result fully justifies the application of high-capacity supervised methods to isolate the genealogical signal. PCA was useful as an exploratory analysis, revealing that part of the variability could be explained by the first two components, although supervised methods are generally more effective for robust classification.

As an unsupervised technique, PCA could not perfectly separate the three groups. Although partial distribution tendencies were observed between Bourbon and Timor Hybrid along PC1, substantial overlap among genealogical groups remained in the PCA space, indicating that the unsupervised model alone was insufficient to promote robust discrimination among classes. Mundo Novo samples were distributed between these groups, with some samples positioned closer to the Timor Hybrid cluster, indicating similarity in chemical profile, whereas others were located closer to Bourbon. This overlap suggests that the differences between Mundo Novo and the other groups may be more subtle or that Mundo Novo exhibits intermediate characteristics. Overall, the variability captured by PC1 and PC2 already indicated relevant distinctions between Bourbon and Timor Hybrid; however, the separation of Mundo Novo was not evident using PCA alone. These findings justify the application of supervised methods to improve discrimination among *Coffea arabica* genealogical groups.

### 3.2. Performance and Validation of Chemometric Models

The performance of the supervised models was evaluated using nested cross-validation, a fundamental strategy to reduce optimistic bias in high-dimensional metabolomic datasets with limited sample size. The evaluated metrics included Overall Accuracy, Balanced Accuracy, and Cohen’s Kappa coefficient, allowing simultaneous assessment of global predictive performance, class balance, and agreement beyond random classification.

**Figure 3:**
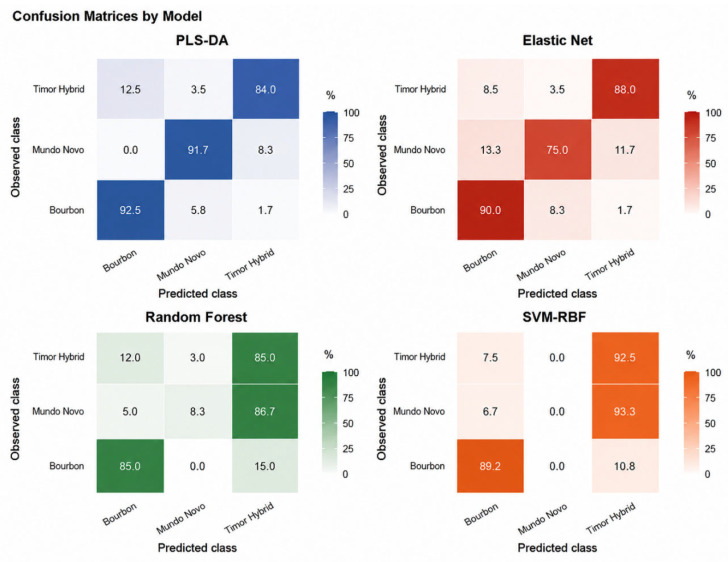
Comparative classification performance of supervised chemometric models applied to the discrimination of *Coffea arabica* genealogical groups based on ^1^H NMR data.

The results demonstrated important differences in the behavior of the algorithms in response to the metabolomic complexity of Coffea arabica genealogical groups (Table 2; Figure 3). Overall, the models showed distinct performances according to their mathematical characteristics and the correlation structure present in the NMR spectral dataset.

Repeated stratified cross-validation demonstrated that the supervised models exhibited distinct performances in response to the metabolomic complexity of *Coffea arabica* genealogical groups. Overall, the results confirmed that discrimination among groups is feasible using ^1^H NMR spectra, although efficiency varied according to the mathematical structure of each algorithm.

The PLS-DA model achieved the highest overall accuracy (87.9%), the highest Balanced Accuracy (89.4%), and the most balanced performance among the three genealogical classes. In addition, the confidence interval associated with Balanced Accuracy indicated moderate to high predictive stability, considering the limited sample size and the high dimensionality of the dataset. The homogeneous behavior of the individual recalls (Bourbon = 92.5%; Mundo Novo = 91.7%; Timor Hybrid = 84.0%) demonstrated that PLS-DA was the least affected by class imbalance, a particularly important characteristic in metabolomic datasets with reduced sample size and uneven class distribution. These results reinforce the suitability of PLS-DA for highly collinear spectral datasets, since the method projects variables into latent components optimized to simultaneously maximize group separation and explained variance.

The Elastic Net model showed overall accuracy very close to that of PLS-DA (86.6%) and a high Kappa coefficient (0.790), indicating agreement between predicted and observed classifications. The good performance of this model suggests that genealogical discrimination may be partially explained by reduced subsets of highly informative spectral variables, since Elastic Net combines L1 and L2 regularization, simultaneously promoting variable selection and control of metabolomic collinearity. The model exhibited particularly high performance for Bourbon (recall = 90.0%) and Timor Hybrid (88.0%). However, the lower performance for Mundo Novo (75.0%) indicates greater metabolomic overlap of this group with the others, especially due to its intermediate nature between Bourbon and Timor Hybrid.

The SVM-RBF model showed intermediate performance, with overall accuracy of 76.8% and Balanced Accuracy of 60.6%. The algorithm demonstrated high ability to discriminate Timor Hybrid (92.5%) and Bourbon (89.2%), suggesting that nonlinear relationships partially contributed to the separation of these groups. However, the extremely low recall for Mundo Novo (0.0%) indicates greater sensitivity of SVM-RBF to intraclass heterogeneity and metabolomic overlap observed in this group. In small and highly correlated datasets, highly flexible nonlinear models may exhibit increased susceptibility to overfitting local classification boundaries, reducing their overall stability.

Random Forest showed the lowest overall performance, with overall accuracy of 72.9% and Balanced Accuracy of 59.4%. Despite the good identification of Timor Hybrid (85.0%) and Bourbon (85.0%), the model exhibited low sensitivity for Mundo Novo (8.3%), indicating substantial difficulty in establishing consistent classification boundaries for this genealogical group. This behavior suggests that the metabolomic structure of the dataset was better represented by linear latent-variable models, favoring projection-based and regularized approaches rather than decision tree-based algorithms. Furthermore, the high dimensionality associated with the small sample size tends to reduce the stability of individual trees, increasing variability in ensemble decisions.

Overall, the results demonstrate that genealogical separation in *Coffea arabica* is mainly associated with gradual and predominantly linear metabolomic patterns, particularly related to lipids, amino acids, trigonelline, caffeine, and chlorogenic acids. The superior performance of PLS-DA and Elastic Net reinforces that regularized linear models provide greater stability and interpretability for metabolomic applications involving ^1^H NMR data. On the other hand, the consistently lower performance observed for Mundo Novo across nearly all models suggests a partially intermediate metabolomic profile, which is consistent with its genetic origin derived from the natural cross between Bourbon and Typica, although the reduced number of samples within the Mundo Novo group may also have contributed to greater classification instability.

Despite the promising results obtained from the supervised models, some limitations should be considered when interpreting the findings. The sample set used in this study is still relatively small for high-dimensional supervised metabolomic approaches, particularly for the Mundo Novo group, which contained fewer samples than the other genealogical groups. Class imbalance may affect model stability and favor classification bias, especially in more complex algorithms sensitive to sample distribution. Nevertheless, the use of robust metrics such as balanced accuracy, combined with repeated cross-validation and nested cross-validation strategies, partially minimized these effects and increased the statistical reliability of the analyses. Even so, future studies involving a larger number of accessions, multiple crop seasons, and independent external validation will be important to confirm the stability of the identified metabolomic markers.

### 3.3. Main Chemical Compounds Responsible for the Separation of Coffea arabica Genealogical Groups

PLS-DA analysis of the ^1^H NMR spectra revealed the main chemical compounds responsible for the separation of *Coffea arabica* genealogical groups. The Bourbon group, associated with negative coefficients, showed higher relative abundance of sucrose, lipids, and trigonelline, compounds related to sweetness, light body, and delicate aroma, which are characteristic of high-quality coffees. The Mundo Novo group, associated with intermediate coefficients, was characterized mainly by the contribution of amino acids and organic acids, which are precursors of aroma and body-related compounds, reflecting a balanced sensory profile between acidity and intensity. In contrast, the Timor Hybrid group, associated with positive coefficients, was characterized by the predominance of trigonelline, chlorogenic acids, and caffeine, phenolic and nitrogen-containing compounds associated with higher bitterness and acidity, which are commonly related to resistant genotypes. It is important to emphasize that these identified compounds represent potential precursors or chemical markers associated with sensory attributes described in the literature, rather than compounds directly determining such attributes.

**Figure 4:**
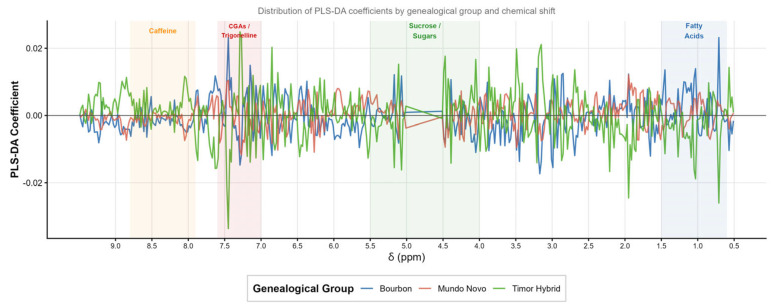
Distribution of coefficients and main chemical regions by 1H NMR PLS-DA.

Figure 4 shows the distribution of chemical annotations across the PLS-DA coefficients: 7.9–8.8 ppm (orange) corresponds to the caffeine region, more strongly associated with Timor Hybrid (positive coefficients); 7.0–7.6 ppm (red) corresponds to trigonelline and chlorogenic acids, which also predominated in Timor Hybrid; 4.0–5.5 ppm (green) corresponds to sucrose and sugars, associated with Bourbon (negative coefficients); and 0.6–1.5 ppm (blue) corresponds to fatty acids and lipids. Therefore, the plot visually demonstrates which spectral regions discriminate against each genealogical group: Bourbon is associated with negative regions (blue and green), Timor Hybrid with positive regions (red and orange), and Mundo Novo occupies an intermediate position between both groups.

**Figure 5:**
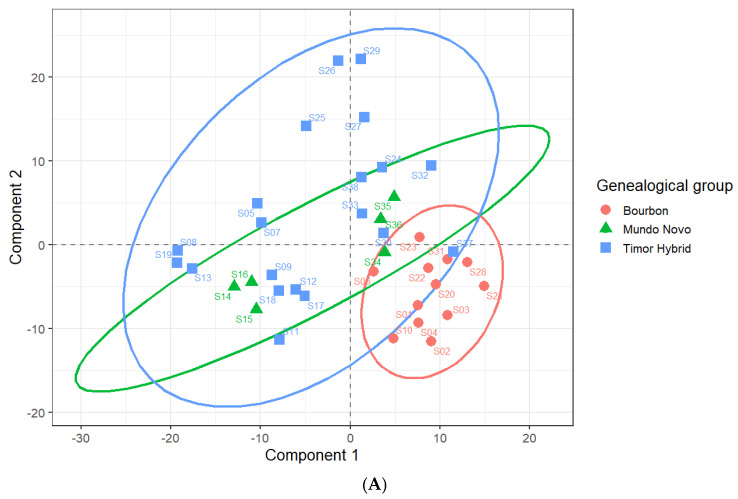

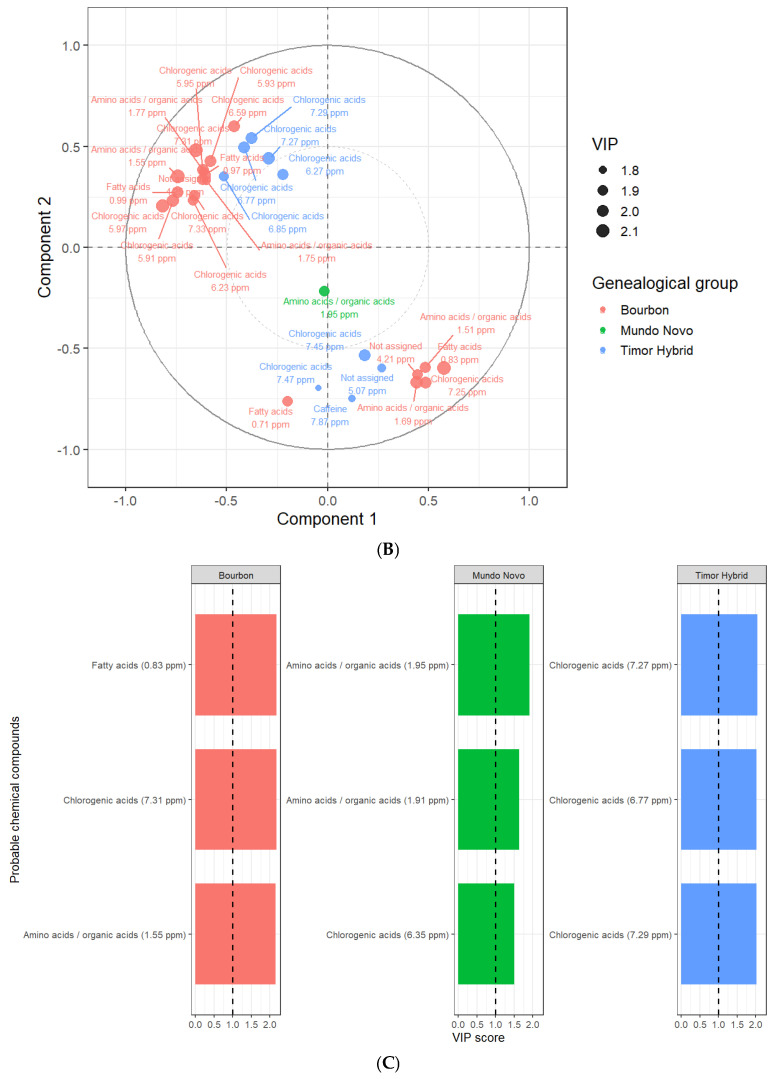

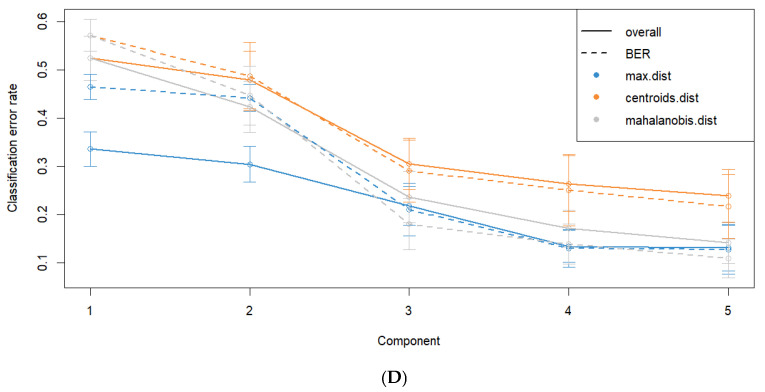
(**A**) Graph of the PLS-DA scores of two components for the chemical composition differentiating the genealogical groups. (**B**) Correlation loading plot of chemical compounds with components 1 and 2 with the classes Bourbon, Mundo Novo and Timor Hybrid. (**C**) Top three discriminant ^1^H NMR buckets associated with each genealogical group based on component 3 of the PLS-DA model. The variables were selected according to the highest absolute loadings in component 3 combined with positive class association. Putative metabolite assignments were based on literature-reported ^1^H NMR spectral regions for coffee metabolites. (**D**) Cross-validated classification error rates for the PLS-DA model according to the number of latent components using repeated stratified 5-fold cross-validation (100 simulations).

Therefore, the PLS-DA classification model was constructed to discriminate the genealogical groups. Figure 5A shows the projection of the accession codes onto the first two PLS-DA components according to the loadings of the corresponding components from matrix X. A certain degree of overlap among groups was still observed, indicating the existence of common metabolomic regions among accession codes, as evidenced by the 95% confidence ellipses shown in Figure 5A.

Figure 5B,C presents the correlation plot and the main discriminant buckets selected from components 2 and 3 of the PLS-DA model, respectively, highlighting the ^1^H NMR spectral regions most associated with the discrimination of genealogical groups. For the Bourbon group, the variables that most influenced classification were lipids, amino acids, and organic acids. In the Mundo Novo group, amino acids and organic acids were also the compounds that most contributed to separation, although with lower intensity. Chlorogenic acids were the variables that most strongly influenced the classification of the Timor Hybrid group.

According to the PLS-DA results, it was possible to identify the variables with the greatest influence on the classification of genealogical groups and, consequently, determine potential chemical markers associated with these genotypes. The chemical compounds present in green coffee beans are important precursors of flavor and aroma compounds formed during the roasting process. Breeding programs aimed at improving beverage quality are complex because the sensory parameters evaluated during cupping are strongly influenced by environmental conditions [17,18]. Therefore, analytical tools capable of identifying chemical markers associated with beverage quality are required [19].

To verify and validate the constructed model, the classification error rate was evaluated (Figure 5D), and the selected error rate corresponded to component 5 (10.5%). It should be emphasized that this error rate was obtained through a repeated 5-fold cross-validation procedure, in which one subset was used to fit the model and the remaining subsets were used for testing across 100 simulations. The permutation test demonstrated that the observed classification performance was significantly superior to that obtained by random assignment of class labels (*p* < 0.05). The real PLS-DA model exhibited a substantially lower balanced error rate than the distribution generated by the permuted models, indicating that the observed discrimination was not due to random chance compared with the distribution generated from random permutation of class labels, corroborating the robustness and non-random nature of the metabolomic discrimination among *Coffea arabica* genealogical groups.

**Figure 6:**
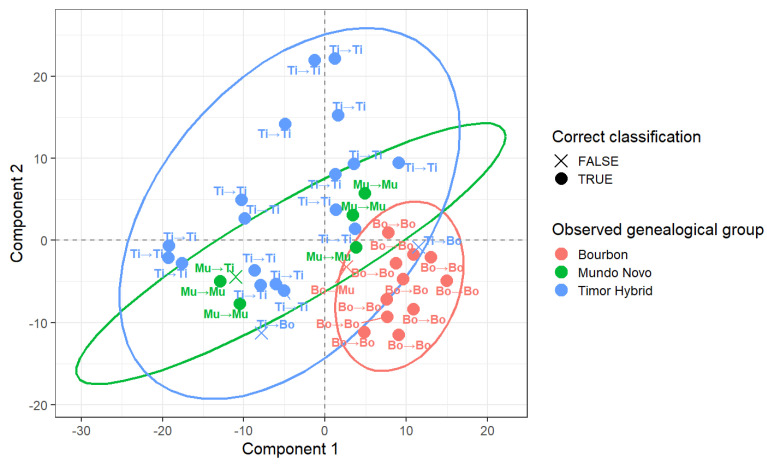
Graph of the PLS-DA scores of five components for the chemical composition with classifications of the genealogical groups. The PLS-DA model (5 components, 5-fold cross-validation × 100 simulations) showed discrimination among the genealogical groups based on the chemical composition of coffee samples.

According to the PLS-DA model, each accession code was classified into the genealogical group presenting the highest loading value in the PLS-DA component associated with the lowest classification error rate (Figure 5D). The PLS-DA model containing five components was selected as the final model because it provided the best balance between predictive performance, chemical interpretability, and statistical parsimony. According to the PLS-DA model fitted with five latent variables, the genealogical groups showed moderate separation with partial overlap among classes (Figure 6). The Bourbon group presented the highest classification consistency, whereas the Timor Hybrid group showed greater dispersion and higher misclassification rates. The overall model performance reached 89.5% accuracy after repeated 5-fold cross-validation (100 simulations), indicating satisfactory discrimination power based on the chemical composition of coffee beans. Thus, Figure 6 shows the classifications resulting from the PLS-DA model, in which one sample from the Bourbon group, one from Mundo Novo, and two from Timor Hybrid were not correctly classified (Figure 6 and Table 3). Therefore, the samples with the highest correct classification rate belonged to the Bourbon genealogical group, which presented the lowest error rate (8.3%). The performance metrics presented in Table 2 correspond to repeated stratified 5-fold cross-validation (100 repetitions) performed after hyperparameter optimization through nested cross-validation, whereas Table 3 summarizes the performance of the final optimized model containing five latent variables.

### 3.4. Metabolic Signatures for Genealogical Discrimination

**Figure 7:**
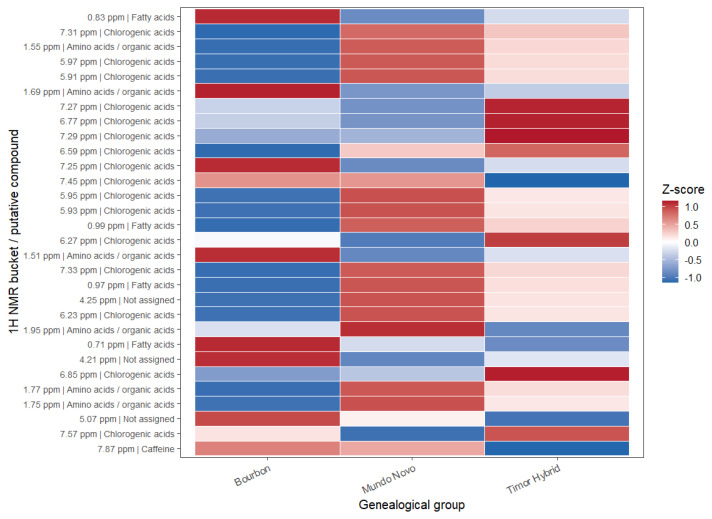
Z-score normalized heatmap of the Top 30 VIP variables by genealogical group obtained from the PLS-DA model. The heatmap displays the thirty most discriminant chemical variables (VIP scores) identified by the PLS-DA model, normalized by Z-score across the three genealogical coffee groups (Bourbon, Mundo Novo, and Timor Hybrid). Red shades indicate higher relative intensities (positive Z-scores), whereas blue shades represent lower relative intensities (negative Z-scores). Each variable is identified by its chemical shift (δ, ppm) and its major compound class detected in the ^1^H NMR spectra. The color distribution highlights the distinct metabolomic profiles characterizing each genealogical group. The Bourbon group showed relatively higher intensities in spectral regions associated with lipids, amino acids, and organic acids, whereas Mundo Novo displayed intermediate intensities. In contrast, Timor Hybrid was enriched in chlorogenic acids and aromatic phenolic compounds.

The analysis of the variables with the highest coefficients in the models (Figure 7) provided strong biochemical plausibility for the separation of the genealogical groups (Table 4).

The identification of discriminant metabolites was performed based on loading plots (PC1 × PC2) and confirmed by bidimensional heteronuclear correlation experiments (^1^H–^13^C HSQC and HMBC). Thus, the assignment of chlorogenic acids, amino acids, organic acids, and lipids was confirmed, whereas additional signals were annotated putatively. The consistency between the identified chemical markers and the genetic background of the samples validates the metabolomic approach.

The integration of PLS-DA coefficients, VIP values, and bidimensional NMR experiments (HSQC and HMBC) allowed the identification of consistent metabolomic signatures associated with the different genealogical groups of *Coffea arabica*. The Bourbon group showed greater association with lipids, organic acids, and amino acids, compounds related to creaminess and aromatic refinement. The Mundo Novo group exhibited an intermediate metabolic profile, mainly characterized by amino acids and organic acids, suggesting a balance between aroma precursors and compounds associated with beverage body. In contrast, the Timor Hybrid group showed higher relative intensities of chlorogenic acids, reflecting a more phenolic and acidic chemical profile associated with its interspecific genetic origin. These results reinforce the biological consistency of the chemometric models and demonstrate the potential of ^1^H NMR metabolomics for genealogical discrimination of *Coffea arabica*.

#### 3.4.1. Chemical Interpretation of the Bourbon Genealogical Group

The signal observed at δ 0.83 ppm is typical of terminal methyl protons (–CH_3_) from aliphatic chains of saturated fatty acids, such as palmitic acid (C_16:0_) and stearic acid (C_18:0_), in addition to possible contributions from triacylglycerols [20,21]. In the ^1^H NMR spectrum, this region (0.7–1.0 ppm) is widely attributed to neutral lipid components of green and roasted coffee.

These compounds are classical chemical markers associated with beverage body and creamy texture, since lipids participate in the retention of volatile compounds and contribute to viscosity perception [22]. The high intensity of this signal in the Bourbon group suggests a higher lipid content compared with other groups, which is consistent with the metabolic profile of *C. arabica*, generally characterized by higher proportions of total oils (~15% dry weight) compared with hybrids related to *C. canephora* [23,24].

From a sensory perspective, higher lipid content may potentially be associated with compounds previously related to enhanced body and aromatic persistence, in addition to attenuating acidity and bitterness perception [25]. Thus, lipid-associated signals may contribute to chemical characteristics previously related to beverage body and creaminess.

The bucket centered at δ 1.69 ppm was putatively associated with overlapping signals from aliphatic amino acids and organic acids located within the 1.6–2.0 ppm region of the ^1^H NMR spectrum [20]. These metabolites play an essential role as precursors of volatile aroma compounds formed during the roasting process. During the Maillard reaction, amino acids react with reducing sugars, generating pyrazines, furanones, and aldehydes responsible for fruity, caramel-like, and floral notes [25,26]. Thus, the significant presence of this signal in the Bourbon group may be associated with characteristics previously related to aromatic potential and sensory complexity in coffees described in the literature as having an intense and pleasant aroma. As no sensory (cupping) analysis was performed in this study, this relationship is presented as a hypothesis to be confirmed rather than as an experimentally measured sensory outcome.

The exact identification of the compounds within this bucket is hindered by signal overlap, an inherent characteristic of NMR analyses of complex mixtures. However, studies using bidimensional spectra (HSQC and HMBC) confirm that this region is predominantly composed of free amino acids and carboxylic acids associated with fruit maturation metabolism [20].

The signal at δ 7.25 ppm is in the aromatic proton region of conjugated phenolic compounds, including chlorogenic acids (CGAs), caffeic acid, and trigonelline derivatives. This region is typical of aromatic phenolic compounds recognized for their antioxidant activity and contribution to acidity and aroma [26]. During roasting, CGAs undergo thermal degradation, generating phenol, guaiacol, vanillin, and cresols, compounds responsible for caramel, vanilla, and chocolate notes [27]. Although the Bourbon group presented lower relative intensities of these compounds compared with Timor Hybrid, their moderate presence contributes to balanced acidity and aromatic complexity without the excessive bitterness commonly associated with resistant hybrids.

#### 3.4.2. Chemical Interpretation of the Mundo Novo Genealogical Group

The Mundo Novo group was mainly discriminated against by amino acids and organic acids. In the PLS-DA model, the Mundo Novo genealogical group showed the most discriminant variables at the chemical shifts δ 1.75, δ 1.95, and δ 7.31 ppm, corresponding to the classes’ amino acids/organic acids and chlorogenic acids/aromatic phenolics. These compounds reflect a balanced and intermediate chemical profile between the Bourbon and Timor Hybrid groups, consistent with the hybrid origin of Mundo Novo, a natural cross between the Typica and Bourbon varieties of *Coffea arabica*.

The signals at δ 1.75 and δ 1.95 ppm are associated with aliphatic amino acids (such as alanine, valine, leucine, and isoleucine) and low-molecular-weight organic acids (such as malic, citric, succinic, and lactic acids), which act as precursors of volatile aroma compounds during roasting [25,26]. During thermal processing, these metabolites participate in Maillard reactions with reducing sugars, forming volatile nitrogen-containing compounds (pyrazines, furanones, and aldehydes) responsible for sweet, floral, and fruity notes [25]. Therefore, the expressive presence of these compounds in the Mundo Novo group suggests metabolically balanced beans with potential for coffees exhibiting medium body and complex aroma, sensory characteristics typically associated with this variety.

On the other hand, the signal at δ 7.31 ppm is characteristic of aromatic protons from chlorogenic acids (CGAs) and other aromatic phenolic compounds. CGAs are the major phenolic compounds present in green coffee and play a central role in defining beverage acidity, bitterness, and antioxidant potential [26]. During roasting, these compounds undergo partial thermal degradation, generating volatile derivatives such as phenol, guaiacol, vanillin, and cresols, which contribute caramel, vanilla, and chocolate notes [27].

The coexistence of amino acids and chlorogenic acids at expressive levels in the Mundo Novo group indicates a chemical balance between aroma precursors and acidity-related compounds, resulting in a balanced sensory profile with good sweetness, medium body, and bright acidity, characteristics frequently observed in coffees from this lineage [19].

In summary, the Mundo Novo group stands out for presenting an intermediate chemical profile between the extremes represented by Bourbon (sweetness and body) and Timor Hybrid (acidity and bitterness), representing a metabolic and sensory balance highly appreciated in specialty coffees.

#### 3.4.3. Chemical Interpretation of the Timor Hybrid Genealogical Group

The Timor Hybrid genealogical group presented, in the PLS-DA model, the most expressive discriminant variables at the chemical shifts δ 7.29, δ 6.85, and δ 6.77 ppm, all located within the aromatic region of the ^1^H NMR spectrum. These signals correspond predominantly to phenolic compounds and chlorogenic acid (CGA) derivatives, which are recognized as chemical markers associated with acidity, bitterness, and antioxidant activity in coffee [26].

Chlorogenic acids are esters formed between quinic acid and caffeic acid and represent one of the major classes of phenolic metabolites in green coffee, accounting for approximately 4–10% of the bean dry matter [28]. The variations observed in the intensity of these spectral regions in Timor Hybrid indicate higher accumulation of CGAs, which is consistent with the genetic background of this group, derived from the cross between *Coffea arabica* and *Coffea canephora* (robusta). This inheritance confers to Timor Hybrid a chemical profile closer to robusta coffee, characterized by higher levels of phenolic and bitter compounds, as well as greater resistance to biotic and abiotic stresses [19,29].

During the roasting process, chlorogenic acids undergo thermal rearrangements and oxidative degradation, generating a variety of volatile aromatic compounds such as phenol, guaiacol, cresols, and vanillin, which are directly responsible for roasted, bitter, and full-bodied beverage notes [24]. However, excessive degradation of these phenolic compounds may also generate reactive quinones associated with increased astringency and residual bitterness.

The predominance of signals in the δ 6.7–7.3 ppm region in Timor Hybrid demonstrates the strong presence of conjugated aromatic structures, typical of caffeic, ferulic, and p-coumaric acids, in addition to trigonelline and nicotinamide derivatives, which also contain active aromatic protons in this region [20]. Trigonelline is a precursor of niacin (vitamin B3) and contributes to sweet and slightly spicy notes after roasting.

This high concentration of aromatic phenolic compounds confers on Timor Hybrid a distinct chemical profile characterized by high acidity, perceptible bitterness, and elevated antioxidant potential. In sensory analyses, coffee with these characteristics usually presents pronounced acidity and intense body, frequently associated with spicy and cocoa-like notes [27].

Thus, the set of observed variables suggests that the Timor Hybrid group is distinguished by the chemical intensity of phenolic compounds, being the group that differs most strongly from the others due to the pronounced presence of chlorogenic acids and aromatic phenolics. This profile reinforces its hybrid genetic origin and explains the behavior observed in the Top 30 heatmap, in which Timor Hybrid exhibited higher Z-scores in the aromatic region of the spectrum.

## 4. Discussion

The present study demonstrated that ^1^H NMR-based metabolomics combined with supervised chemometric models can discriminate three genealogical groups of *Coffea arabica*—Bourbon, Mundo Novo, and Timor Hybrid—based on their metabolic fingerprints. These findings are consistent with the hypothesis that genealogical signals persist in the NMR metabolome despite environmental variation introduced by crop season, altitude, and agronomic management, as pointed out by Bollen et al. [13] and de León-Solis et al. [10].

The inability of PCA to clearly separate the three genealogical groups aligns with previous reports in the coffee metabolomics literature. The strong G × E interaction in *C. arabica* introduces considerable metabolic variability that dilutes the genealogical signal in unsupervised analyses [11,16]. Similar observations were reported by Monakhova et al. [6] and Villálon-López et al. [7], who noted that NMR-based PCA of coffee extracts typically captures post-harvest and environmental variation more strongly than genotypic differences. The cumulative variance of 52.91% explained by PC1 and PC2 is consistent with the high spectral complexity of green coffee extracts, which contain hundreds of overlapping metabolites.

Among the supervised models, PLS-DA outperformed Elastic Net, SVM-RBF, and Random Forest in balanced accuracy (89.4%) and class-level recall homogeneity. This result is consistent with the well-established suitability of PLS-DA for high-dimensional, highly collinear metabolomic datasets obtained by NMR [8]. The latent-variable projection in PLS-DA simultaneously maximizes explained variance and group separation, a mathematical property that is particularly advantageous when the number of variables greatly exceeds the number of samples, as is the case here (425 buckets vs. 38 samples). The similar performance of Elastic Net (86.6% overall accuracy) corroborates the finding that genealogical discrimination can be partly captured by a sparse subset of highly informative spectral variables, since Elastic Net combines L1 and L2 regularization to perform simultaneous variable selection and collinearity control [12].

The consistently low recall for Mundo Novo across all models—reaching 0% in SVM-RBF and 8.3% in Random Forest—reflects the metabolic intermediacy of this group, which originated from a natural cross between the Bourbon and Typica varieties [14]. The intermediate positioning of Mundo Novo between Bourbon and Timor Hybrid in the PLS-DA score plots is biologically coherent with its genetic origin, and this pattern has also been reported in studies using different analytical platforms for coffee varietal characterization [2,5]. Importantly, the reduced number of Mundo Novo samples (n = 6) likely amplified classification instability in tree-based and nonlinear models, which are more sensitive to class imbalance than regularized linear methods. Expanding the Mundo Novo sample set in future studies would be essential to verify whether the intermediate metabolic profile reflects genuine genotypic characteristics or a consequence of limited sampling.

The metabolic signatures identified by VIP scores and PLS-DA loadings are biochemically coherent with the genetic background of each genealogical group. The higher relative abundance of lipids in Bourbon is consistent with the general lipid profile of *C. arabica*, which typically presents approximately 15% total oil content on a dry weight basis, higher than interspecific hybrids involving *C. canephora* [23,24]. Lipids, particularly triacylglycerols and free fatty acids, contribute to beverage body and the retention of volatile aroma compounds, and their enrichment in Bourbon may partly explain the sensory attributes of smoothness and aromatic persistence frequently described for coffees of this lineage [22,25].

The enrichment of chlorogenic acids (CGAs) in Timor Hybrid aligns with the known higher phenolic content of C. canephora-derived germplasm [19,29]. CGAs in green coffee account for approximately 4–10% of bean dry matter [28] and are key precursors of bitter and acidic taste compounds generated during roasting [26]. The predominance of CGA-related signals in Timor Hybrid, particularly in the δ 6.7–7.3 ppm aromatic region, is consistent with the robusta-like chemical inheritance that characterizes HdT-derived genotypes. This finding is also in agreement with Setotaw et al. [15], who reported that resistance genes introgressed from *C. canephora* into Timor Hybrid lineages are frequently accompanied by altered secondary metabolite profiles, including elevated phenolic compound levels.

The amino acids and organic acids identified as discriminant variables in both Bourbon and Mundo Novo groups are recognized as key Maillard reaction precursors. During roasting, free amino acids react with reducing sugars to produce pyrazines, furanones, and aldehydes responsible for fruity, caramel-like, and floral aromatic notes [25,26]. The co-occurrence of these compound classes with moderate CGA levels in Mundo Novo suggests a chemical balance between aroma precursors and acidity-related compounds, which is consistent with the balanced sensory profile frequently associated with this cultivar in specialty coffee evaluations [17,19].

The structural confirmation of discriminant metabolites through 2D NMR experiments (HSQC and HMBC) reinforces the reliability of the proposed chemical markers beyond the inherent limitations of bucket-based spectral annotation. This approach is consistent with the analytical rigor recommended for metabolomic studies in which signal overlap in complex matrices may hinder unambiguous assignment from 1D spectra alone [20]. The agreement between chemometric models and structural NMR data strengthens the biological consistency of the identified signatures and supports their use as potential molecular markers in varietal traceability systems.

From a practical standpoint, the integration of ^1^H NMR and PLS-DA demonstrated a capability for varietal discrimination compatible with applications in specialty coffee certification and breeding support, as proposed by Malta et al. [4] and Mannino et al. [12]. The relatively non-destructive nature of NMR, combined with its ability to simultaneously profile multiple compound classes in a single acquisition, makes this platform attractive for reference characterization of germplasm bank accessions, such as those of the EPAMIG Active Germplasm Bank used in this study. However, it is important to emphasize that the identified metabolic signatures represent potential chemical markers associated with genealogical groups rather than definitive diagnostic features, given that environmental modulation of the coffee metabolome remains a substantial confounding factor [3,13].

It is also relevant to position ^1^H NMR with respect to other analytical platforms that have been applied to coffee discrimination and authentication. Mass spectrometry-based metabolomics and DNA fingerprinting approaches (e.g., RFLP and SSR markers) provide high sensitivity and, in the case of DNA, direct genotypic information, but generally require more complex sample preparation or specialized expertise. Vibrational spectroscopy techniques such as Fourier-transform infrared (FT-IR) and near-infrared (NIR) spectroscopy, as well as laser-induced breakdown spectroscopy (LIBS), are considerably simpler, faster, and less expensive, and when coupled with PLS-DA they can achieve classification performances comparable to those reported here. These characteristics make them strong candidates for high-throughput field or industrial screening. In this context, the balanced accuracy of 89.4% obtained in the present study with ^1^H NMR and PLS-DA is comparable to discrimination performances previously reported for coffee genotypes and origins using mass spectrometry, vibrational spectroscopy, and classical chemical composition analyses [2,4,6,7,12]. The main advantage of ^1^H NMR is not throughput or cost but rather its high structural resolution and reproducibility, which allow the simultaneous quantification and unambiguous structural assignment of multiple metabolite classes in a single acquisition. Therefore, the role of ^1^H NMR is best understood as a high-resolution laboratory reference method, suitable for marker discovery and metabolite identification, rather than as a routine high-throughput tool. A realistic workflow would employ ^1^H NMR to identify and validate discriminant chemical markers, which could subsequently be transferred to faster and lower-cost techniques (such as FT-IR, NIR, or LIBS) for routine varietal traceability and breeding screening, given the well-recognized logistical constraints associated with the routine use of NMR in high-throughput contexts.

Several limitations should be acknowledged. The dataset is relatively small, particularly for Mundo Novo (n = 6), which constrains the generalizability of the classification models and may inflate performance estimates for this class. Although the use of repeated stratified cross-validation with 100 simulations and nested cross-validation for hyperparameter tuning partially mitigated overfitting risk, external validation with an independent sample set from different environments and crop seasons would be required to confirm the robustness of the proposed markers. Additionally, the restriction to two crop seasons at a single experimental site (EPAMIG, Patrocínio) limits the capacity to disentangle genotypic signals from site-specific environmental effects. Future studies should incorporate samples from multiple locations and growing seasons, expand the number of accessions per genealogical group, and consider integrative approaches combining metabolomic, genomic, and sensory data to increase the discriminatory power and practical applicability of varietal traceability systems for *C. arabica* [10,12,18]. In addition, the natural (dry) processing used to obtain the samples inherently involves a fermentation phase associated with the resident microbiota during fruit drying, which constitutes a potential confounding factor. Because all samples were processed identically, this effect was minimized as a systematic factor across groups; nevertheless, the present data cannot fully disentangle whether part of the discriminant metabolites derives from the plant genotype or from processing-related (microbial) metabolism, and this should be addressed in future studies, ideally comparing different processing methods. It should also be emphasized that no sensory (cupping) analysis was performed in this study; therefore, the associations drawn between the discriminant metabolites and sensory attributes (such as body, sweetness, acidity, and bitterness) are based exclusively on relationships previously reported in the literature and represent hypotheses to be confirmed, rather than experimentally measured sensory outcomes. Likewise, the discriminant compounds were determined in green (unroasted) beans, whereas the final aroma and flavor of the beverage develop through complex thermally driven reactions during roasting (Maillard reaction, Strecker degradation, and chlorogenic acid thermal degradation) whose extent depends strongly on roasting degree. Consequently, the green-bean metabolic profiles reported here should be regarded as precursors and candidate markers rather than direct predictors of the final roasted aroma profile, and explicit sensory validation across controlled roasting levels is recommended in future work.

## 5. Conclusions

The integration of ^1^H NMR metabolomics and chemometric modeling demonstrated promising performance for the discrimination of *Coffea arabica* genealogical groups based on their metabolic profiles. Among the evaluated models, PLS-DA presented the best compromise between classification performance and chemical interpretability, whereas Elastic Net reinforced the robustness of the identified discriminant variables. The metabolic differentiation among Bourbon, Mundo Novo, and Timor Hybrid groups was mainly associated with variations in the relative levels of lipids, amino acids, organic acids, and chlorogenic acids, reflecting biochemical characteristics related to their distinct genetic backgrounds as modulated by genotype × environment interactions. The agreement among the chemometric models and the structural confirmation obtained through bidimensional NMR reinforced the biological consistency of the proposed markers. These results demonstrate the potential of integrating ^1^H NMR and machine learning for genealogical discrimination of *C. arabica*. Rather than a routine high-throughput tool, ^1^H NMR is best positioned as a high-resolution laboratory reference method for the discovery and structural validation of candidate chemical markers, which could subsequently be transferred to faster and lower-cost techniques (such as FT-IR, NIR, or LIBS) to support varietal traceability and specialty coffee breeding programs. These findings should be confirmed by future studies including independent external validation, multiple environments and crop seasons, and sensory analysis.

## Figures and Tables

**Table 1:** Number of *C. arabica* samples analyzed by genealogical groups and harvest year.

Genealogical Group	Harvest 2020	Harvest 2021	Total
Bourbon	6	6	12
Mundo Novo	3	3	6
Timor Hybrid (HdT)	10	10	20
Total	19	19	38

**Table 2:** Classification performance of supervised models (NMR) by genealogical group of *Coffea arabica*.

Model	Overall Accuracy	Balanced Accuracy	Kappa	Bourbon Recall	MN Recall	Hdt Recall
PLS-DA	0.879	0.894	0.803	0.925	0.917	0.840
Elastic Net	0.866	0.843	0.779	0.900	0.750	0.880
SVM-RBF	0.768	0.606	0.574	0.892	0.000	0.925
R Forest	0.729	0.594	0.512	0.850	0.083	0.850

**Table 3:** Counting false positives and negatives in relation to the classification of the genealogical groups by the PLS-DA model and their respective error rates obtained through repeated 5-fold cross-validation (100 simulations). Performance of the PLS-DA model fitted using five latent variables.

Genealogical Group Observed	Correct	Misclassified	Error Rate (%)
Bourbon	11	1	8.3
Mundo Novo	5	1	16.7
Timor Hybrid	18	2	10
Total	34	4	10.5
Accuracy			89.5

The PLS-DA model with five components demonstrated high classification accuracy for the Bourbon and Timor Hybrid, whereas Mundo Novo exhibited the highest misclassification rate. Overall, the model showed relative consistent and robust performance across 100 cross-validation simulations, indicating a satisfactory ability to discriminate genealogical groups based on chemical composition. The values presented in Table 3 correspond to the optimized final model and therefore are not directly comparable to the repeated cross-validation estimates presented in Table 2.

**Table 4:** Main discriminant metabolites identified by ^1^H NMR and their association with *Coffea arabica* genealogical groups based on PLS-DA analysis.

Genealogical Group	δ (ppm)	Chemical Class/Putative Compound	Possible Chemical Relevance	Interpretation
Bourbon	0.83	Fatty acids/Lipids	Body and creaminess	Associated with higher lipid content, contributing to smooth mouthfeel and persistence
Bourbon	1.69	Amino acids/Organic acids	Aroma precursors	Related to Maillard reaction precursors and sweet/floral aromatic notes
Bourbon	7.25	Chlorogenic acids/Aromatic phenolics	Acidity and antioxidant activity	Contributes to balanced acidity and aromatic complexity
Mundo Novo	1.75	Amino acids/Organic acids	Aroma precursor	Associated with metabolic balance and moderate aromatic complexity
Mundo Novo	1.95	Amino acids/Organic acids	Aroma precursor	Related to body, sweetness and balanced sensory attributes
Mundo Novo	7.31	Chlorogenic acids/Aromatic phenolics	Acidity and antioxidant activity	Contributes to brightness and moderate bitterness
Timor Hybrid	6.77	Chlorogenic acids/Aromatic phenolics	Phenolic compounds	Related to intense body and roasted notes
Timor Hybrid	6.85	Chlorogenic acids/Aromatic phenolics	Aroma precursor	Associated with aromatic intensity and phenolic profile
Timor Hybrid	7.29	Chlorogenic acids/Aromatic phenolics	Acidity and antioxidant activity	Contributes to pronounced acidity and bitterness

## Data Availability

The original contributions presented in this study are included in the article. Further inquiries can be directed to the corresponding author.
